# A scoping study on task shifting; the case of Uganda

**DOI:** 10.1186/1472-6963-14-184

**Published:** 2014-04-23

**Authors:** Sebastian Olikira Baine, Arabat Kasangaki

**Affiliations:** 1 School of Public Health, College of Health Sciences, Makerere University, P. O. Box 7072, Kampala, Uganda

**Keywords:** Task shifting, Lower health worker cadre, Well trained health worker, Competencies, Overload, Support supervision, Policy and guidelines

## Abstract

**Background:**

Task shifting has been implemented in Uganda for decades with little documentation. This study’s objectives were to; gather evidence on task-shifting experiences in Uganda, establish its acceptability and perceptions among health managers and policymakers, and make recommendations.

**Methods:**

This was a qualitative study. Data collection involved; review of published and gray literature, and key informant interviews of stakeholders in health policy and decision making in Uganda. Data was analyzed by thematic content analysis.

**Results:**

Task shifting was the mainstay of health service delivery in Uganda. Lower cadre of health workers performed duties of specialized health workers. However, Uganda has no task shifting policy and guidelines, and task shifting was practiced informally.

Lower cadre of health workers were deemed to be incompetent to handle shifted roles and already overworked, and support supervision was poor.

Advocates of task shifting argued that lower cadre of health workers already performed the roles of highly trained health workers. They needed a supporting policy and support supervision.

Opponents argued that lower cadre of health workers were; incompetent, overworked, and task shifting was more expensive than recruiting appropriately trained health workers.

**Conclusions:**

Task shifting was unacceptable to most health managers and policy makers because lower cadres of health workers were; incompetent, overworked and support supervision was poor. Recruitment of existing unemployed well trained health workers, implementation of human resource motivation and retention strategies, and government sponsored graduates to work for a defined mandatory period of time were recommended.

## Background

The shortage of well-trained health workers is a global dilemma not limited to, but worst, in low and middle income countries (including Uganda). The problem is so acute that staffing levels are as low as 50% or less for certain types of health workers according to the established positions especially in hard to reach and stay rural areas [1]. Shortage of health workers in Uganda has affected the delivery of health services.

Task shifting has been going on in Uganda for a long time. Nurses in Uganda undertake a range of tasks that were formerly the responsibility of doctors, for example, provision antiretroviral therapy. As a result Uganda has expanded its human resources for delivering Human Immune Deficiency Virus/Acquired Immune Deficiency Syndrome services. The health workers received specific training for the tasks assigned to them prior to performing them [2,3]. It has been promoted in the intermittent preventive treatment of malaria in pregnancy [4], home-based management of malaria [5], family planning services [6,7], and in Human Immune Deficiency Virus/Acquired Immune Deficiency Syndrome patient assessment and antiretroviral treatment [8], and treatment of tuberculosis with directly observed short therapy. Nurses, midwives and clinical officers also prescribe drugs, and carry out circumcision, incision and drainage, surgical toilet, eye cataract removal, set up intra-venous drips, etc.

Task shifting was conceived as a mechanism to efficiently and effectively utilise the existing health work force to deliver the needed health services. It involved shifting tasks from;

• doctors, specialised physicians to non-physician clinicians,

• non-physician clinicians to nurses/midwives, and

• Nurses/midwives to nursing assistants.

The World Health Organization provided global recommendations and guidelines to help countries to implement task shifting. They included; adopting task shifting as a public health initiative, creating an enabling regulatory environment for implementation, ensuring quality of care, ensuring sustainability, and the organization of clinical care services [9]. Adhering to World Health Organization recommendations, however, was perceived to make task shifting became more expensive than recruiting new health workers in terms of time and other resources required to prepare health workers to perform duties delegated to them if quality was not to be compromised.

### Health service delivery organization in Uganda

In Uganda, health services are provided by the public (government) and private sectors. The private health sector comprises of the Private-Not-for-Profit organizations (largely faith-based organizations), Private Health Practitioners (or Private for Profit health providers) and the Traditional and Complementary Medicine Practitioners. About 75 percent of the faith-based health facilities exist under four umbrella organizations i.e. Uganda Catholic Medical Bureau, the Uganda Protestant Medical Bureau, the Uganda Orthodox Medical Bureau and the Uganda Muslim Medical Bureau. This study excluded the Complementary Medicine Practitioners. These include indigenous traditional or complimentary practitioners such as the practitioners of Chinese and Ayurvedic medicine although reports showed that about 60 percent of Ugandans actually sought health care from Complementary Medicine Practitioners especially for minor illnesses [10].

The public health sector in Uganda is organized in a hierarchy effectively starting from Health centre IIs to general hospitals (formerly called district hospitals), then the regional referral hospitals, and the national referral hospitals. Below the general hospitals are the Health sub-District health centers. Regional Referral Hospitals and National Referral Hospital were semi-autonomous institutions, they were only financial autonomous. Human resources, health information management system, governance, pharmaceutical, vaccines and equipment/logistics were still centralized. District health services and general hospitals are managed by local governments.

Health services in Uganda are delivered within the framework of decentralization policy. In 1995, the Government of Uganda decentralized delivery of services in order to improve administrative oversight and service delivery. Local governments were empowered to appoint and deploy public servants including health workers within the districts through the District Service Commission. Local governments also planned and oversee service delivery within the districts (Constitution of the Republic of Uganda 1995 (Uganda, [11]) and the Local Government Act 1997 (Uganda, [12]).

### Human resources for health

The Human Resources for Health shortage in Uganda is characterized by: inadequate numbers and skill mixes of the health work force; low motivation, attraction and retention; high attrition rate; and high rate of absenteeism and poor performance.

At the national level, the proportion of approved positions in the public sector filled by trained health professionals was 56% during the fiscal year 2010/2011. Midwives filled 67%, nurses 58%; clinical officers filled 66%, health assistants 61%, Radiographers 52%, doctors 52%, and pharmacists 28% of the established norms.

Uganda’s health sector staffing situation is below the established norms. The proportion of approved positions in the public sector filled by trained health personnel as at October 2010 is shown in Table 1. It shows a grossly inadequate health workforce especially with doctors, pharmacists, dispensers, and laboratory scientists. A large proportion of the available health workforce is urban based while the majority of the Ugandan poor population World Health Organization need a large proportion of health services is rural based.

**Table 1:** Approved and filled positions by trained personnel in the public health sector, October 2010

**Cadre of staff**	**Mulago Hospital**		**Butabika Hospital**		**Regional Referral Hospitals**		**District Health Offices**		**District**		**Total**	**Total**	**%**
									**Health Units**		**Norms**	**filled**	**filled**
	**Norms**	**Filled**	**Norms**	**Filled**	**Norms**	**Filled**	**Norms**	**Filled**	**Norms**	**Filled**			
Doctors	241	203	26	15	520	204	80	63	824	306	1,691	791	52%
Clinical officers	45	56	12	14	395	261	0	5	2,598	1,678	3,050	2,014	66%
Nurses	940	846	154	127	1,371	1,102	80	10	9,098	4,721	11643	6,806	58%
Midwives	121	95	0	0	701	477	0	0	4,536	3,002	5,358	3,574	67%
Pharmacists	8	4	2	2	36	13	0	2	40	3	86	24	28%
Dispensers	34	26	5	5	80	36	0	0	244	78	363	145	40%
Lab. scientists	63	55	6	6	180	108	0	1	2,236	958	2,485	1,128	45%
Radiographers	33	28	2	3	53	35	0	0	80	22	168	88	52%
Health assistants	0	0	0	0	0	0	0	0	2,573	1,570	2,573	1,570	61%
Other health related staff	252	168	87	92	356	173	320	210	4,951	1,816	5,966	2,459	41%
**Grand total**	**1,737**	**1,481**	**294**	**264**	**3,692**	**2,409**	**480**	**291**	**27,180**	**14,154**	**33,383**	**18,599**	**56%**

Source: Ministry of Health (March, 2011).

### Literature review

The health workforce crisis is a global issue. Known causes broadly include; failure to develop, employ/recruit and retain health workers. These are also due to complex relation to the economy and lack of adequate funds, poor work conditions/environment, migration health workers within countries (e.g. to the private sector and non-governmental organizations) and to other countries in search of better packages, and deaths of the existing health workers. The health work force falls far short of required minimum [13].

World Health Organization [14] reported an absolute shortage of 2.3 million physicians, nurses and midwives in 37 Sub-Saharan African countries. The World Health statistics 2009 revealed a global average number of thirteen (13) physicians per 10,000 people. There was a wide disparity between the different regions with the European Region with an average of thirty two (32) physicians per 10,000 population while the African Region had an average of two (2) physicians per 10,000 population [15]. This implied a severe shortage of health professionals to deliver basic essential health services in some of these countries.

The existing pool of skilled health workers was also unevenly distributed, with high concentrations in urban areas and many working in the private sector rather than in public health sector. Efficient and equitable distribution of Human Resources for Health is a critical limiting factor in health service delivery. The uneven distribution of health workers was indicated as one of the limiting factors to the achievement of the Millennium Development Goals [16].

Task shifting was perceived as a measure to mitigate Human Resources for Health shortage crisis and was seen to offer opportunities such as; increased access to life-saving treatment, improved the health workforce skills and mix, and improved health-system efficiency with cost advantages. In this context, task shifting became an alternative to mitigate the shortage of skilled Human Resources for Health in the poor countries [17,18]. However, the degree to which different cadres of health workers could be substituted was arguably limited [19] and caution must be taken to ensure quality and health outcomes are not compromised.

Analysis of primary data collected from five countries; Brazil, Ethiopia, Malawi, Namibia, and Uganda revealed that delegation of specific tasks to community health workers with limited training was associated with increased access to HIV services, particularly in rural areas and among underserved communities, and improved quality of care for HIV. Task delegation to Community Health Workers was also associated with a significant contribution to the delivery of a wide range of other health services. In both Malawi and Uganda, the basic care package for people living with Human Immune Deficiency Virus/Acquired Immune Deficiency Syndrome was designed to be delivered by non-specialist doctors or nurses supported by Community Health Workers and people living with Human Immune Deficiency Virus/Acquired Immune Deficiency Syndrome [20].

Task shifting was perceived as an essential part of any efforts to: achieve universal access to HIV/AID prevention, treatment and care; deliver services to under-served areas; and to sustainably strengthen primary health care services. Reports showed that a lot of task shifting occurred in the wrong direction where the untrained workforces delivered care outside their scope (Global health workforce alliance - FinalReport_ForumSurvey_28Sep09, [21]).

Task shifting was increasingly being promoted as a coping mechanism for general and specific human resource for health shortages. In almost all countries with Non-Physician Clinicians were reported to play prominent roles in Human Immune Deficiency Virus/Acquired Immune Deficiency Syndrome treatment programmes. Malawi, Ethiopia, Tanzania, Zambia, and Uganda built antiretroviral treatment strategies around Non-Physician Clinicians [22].

A systemic analysis of the literature on an effective strategy for addressing shortages of Human Resources for Health in HIV treatment and care revealed that task shifting offers high-quality, cost-effective care to more patients than a physician-centred model. The main challenges include; adequate and sustainable training, support and pay for staff in new roles, integration of new members into healthcare teams, and compliance of regulatory bodies. It was argued that task shifting be considered when Human Resources for Health shortages threaten programme rollout [23,24].

Low job satisfaction and motivation reduce performance and constitute a significant push factor for migration of health workers from; rural to urban areas, public to private sector, and to other countries. The major job satisfiers include; manageable workload, staff deployment that match with their skills and experience, appropriate compensation, support supervision and job security. Retention factors include; good working and living conditions, opportunities for continuing professional development and career progression, accessibility to good schools for the children of health workers in close proximity to the health facility, and participation in leadership and management. Lack of logistics; drugs, equipment and supplies were observed to cause redundancy and migration to other places of work. These factors were perceived to constitute positive and conducive work climate (Capacity Project [25]).

The working environment has a strong influence on job satisfaction. Poor organisation and environment management are a strong de-motivating force. Decisions by health workers to migrate are usually related to a poor working environment (Ministry of Health, October [26]).

Task-shifting required standardized protocols that include simplified clinical guidelines, recording and reporting systems, and monitoring and evaluation. Definition of the scope of work was required to allow cadres to carry out job functions normally performed by higher level cadres, and to avoid untrained workforce delivering care outside their scope with associated complications, costs and legal implications. This facilitates delegation of interventions to lower level health worker cadres and ensures that quality of care is not compromised while it improves access, increases coverage and geographical equity [20].

Critical to successful implementation of task shifting were perceived to be; existence of political will and commitment, definition of scope of the work for each cadre of health worker, mentoring and support supervision of lower health worker cadres receiving delegated roles, continuous education, adequate remuneration of health workers for the additional workload, and performance evaluation of health workers [3].

### Statement of the problem

The human resource for health crisis in Uganda is characterised by inadequacy in-terms of numbers and skills, and failure to effectively meet the health needs of the Ugandan population. Task shifting has been practiced in Uganda for a long time but there is little documented evidence available on it. It is questionable whether task shifting is acceptable, implemented according to World Health Organization guidelines and recommendations, and how it is perceived among the health policy makers and health care managers, and its impacts.

### Objectives of the study

The objectives of this study were;

1. To provide a synthesis of the available evidence about task-shifting experiences in Uganda.

2. To establish levels of acceptability and perceptions of task shifting among health care managers and policymakers, and

3. To provide recommendations on the implications of Task shifting for human resource management policy.

## Methods

### Study design

This was a qualitative study. Qualitative research was applied in order examine, reflect and understand task shifting in the peculiar Ugandan context.

### Study population

This was a purposive qualitative study whose participants were drawn from the national and district levels. At the national level, key informants interviewed were health policy and decision makers drawn from the Ministry of Health, Uganda Catholic Medical Bureau, Uganda Protestant Medical Bureau, Uganda Muslim Medical Bureau, World Health Organization Uganda Country office, Uganda National Health Consumers Organisation, Uganda Medical and Dental Practitioners Council, Uganda Dental Association, Uganda Nurses and Midwives Council, Uganda Allied Professional Council, and Capacity Program Uganda. Key informants interviewed at the district level included; District Health Officer, and the Resident District Commissioner.

This scoping study only targeted key stakeholders or organizations that are involved in policy formulation. Only representatives of these organizations participated in data collection. Participants were selected purposively and priority was given to those in leadership positions first. No statistical formula was used to obtain the sample size. Motivation for the sample selection methods used were; this was a scoping study to generate in-depth evidence from the key stakeholders in health policy formulation especially human resources for health; and a purposive sample would provide the required information on which a comprehensive study would be developed. This scoping study targeted policy makers and did not include health workers at the health facilities. Consumers of health services were represented by a representative of the people and Civil Society Organizations.

### Data collection

Data collection was a three stage process. Firstly, published and gray literature was reviewed. Information on human resources for health was obtained from; documents in the Ministry of Health, journals and different electronic data bases such as PubMed, Cochrane Library, and Social Science Citation Index were searched.

Secondly, key informant interviews were conducted to seek clarification on specific issues on task shifting. Open-ended questions were used to investigate issues on task shifting and obtain in-depth information on task shifting. Participants were from a wide and varied background but stakeholders in the delivery of health services. Selected key informants held high positions in their organizations included in the sample. They provided their day to day knowledge and experiences on task shifting.

Thirdly, a group discussion of 16 representatives drawn from the Ministry of Health, Development Partners, Professional bodies, Civil Society Organizations/Non-governmental organizations, and the academia was conducted to gain consensus on the issues raised during document review and key informant interviews. Focus group discussions were not conducted.

### Data analysis

Thematic content analysis was used to analyse qualitative data. The process of analysis was iterative. Tape recorded interviews were transcribed, and the information combined with the responses recorded by note-taking. Initial familiarization with the data was done at this stage. Multiple-coding involving cross-checking of coding strategies and interpretation of data by different researchers was applied. The themes included: shortage of health workers; policy and legal framework, recruitment, deployment and retention of trained health workers; support supervision; training of health workers; nursing aides; village health teams; and induction in relation to task shifting that is perceived as means to address human for health issues.

Two researchers were involved in this process. Multiple coding was done to create coding categories that reflect the content of the data and concepts used by the informants rather than the questions in the interview guide. The coding categories extracted from the transcripts were used to systematically analyse commonalities and apparent contradictions reflected in the data by focusing on issues that were repeatedly mentioned or strongly emphasised.

### Ethical considerations

The research protocol was submitted for consideration, guidance and approval to the Uganda National Council of Science and Technology (reference number SS 2444). Uganda National Council of Science and Technology takes into consideration the laws and regulations governing research in Uganda. Uganda National Council of Science and Technology approves and monitors ongoing studies regularly. It is independent of the researchers, sponsors and any other undue influence.

Each potential subject was adequately informed of the aims, methods, sources of funding, institutional affiliation of the researchers, and the anticipated benefits and potential risks of the study. There was no conflict of interest in this research study. Subjects were assured that there would be no harm as a consequence of participation in this research study or predictable risks and burdens to the individuals and communities in comparison with foreseeable benefits to them and to other individuals or communities.

The potential subjects were informed of their right to refuse to participate in the study or to withdraw consent to participate at any time without reprisal. Precautions were taken to protect the privacy of the subjects, confidentiality of personal information, and to minimize the impact of the study on their physical, mental and social integrity. Subjects gave informed consent to participate in this research study without duress. They were also informed of their freedom to access the results of this study and to use evidence generated by this study as they wished.

### Limitations to this scoping study

1. Health providers at the implementation level were not adequately represented to give their views on task shifting. This was planned for the broader and comprehensive study that was to follow this.

2. This was a qualitative study and rigorous statistical analysis could not be done.

3. Participants in this scoping study were drawn from the policy making circle and one district. The views obtained from one district may not represent the views of the other districts in Uganda.

4. The study had limited funding support.

## Results

Almost all task-shifting reported has been taken in preventive services and none in surgical services.

This section presents key findings from selected respondents at the district and national levels. There was a consensus among all key informants that there is a critical shortage of Human Resources for Health in Uganda that needed to be addressed as a matter of urgency. Documents reviewed showed the magnitude of the Human Resources for Health shortage (see Table 1).

Policy makers acknowledged the shortage of health workers in Uganda. A respondent said, "Yes it is indeed true that Human Resources for Health are in great shortage. Of the staffing norms in the public health facilities, only about 54% were filled by the time of this study. The human resources manual shows that doctors make up about 3% (which is really painful)". Nursing Assistants still make up a great percentage (about 17%) of the health work force.

Task shifting was the mainstay of health service delivery in Uganda for a long time. Tasks were shifted from different cadres of different specialities at high ranks to cadres at lower ranks. Tasks were shifted to clinical officers, nurses/mid-wives, laboratory assistants/microscopists, public dental health officers, anaesthetic assistants, and nursing aids. Task-shifting was applied as a coping mechanism to address Human Resources for Health shortage.

One respondent revealed that "clinical officers handle post abortion cases and minor surgery, nurses give anaesthesia to patients undergoing surgery and do medical male circumcision but one must be careful when delegating roles for fear of legal implications in the case of complications".

The rate of shifting tasks far outweighs number of individuals who qualify to receive new additional roles. Health workers were not always deployed in relation to their training or area of specialisation. Overall, task shifting is not easy to implement according World Health Organization guidelines and recommendations.

### Causes of shortage of health workers

Respondents concurred that shortage of health workers was due to the following;

a. Inadequate recruitment of Human Resources for Health in terms of numbers and skills. There were well trained health workers who were unemployed but could not be absorbed by public service because of budget ceilings/limitations.

b. Poor remuneration of health workers de-motivates them. Promises to increase salaries for health workers were endless but were not honoured. Health workers could not afford basic human needs. Some health workers opted out the public service to do private practice or migrate to other organisations or other countries in search for better reward packages or even do non-medical business to generate better income for their families.

c. Inequitable remuneration of health workers in relation to other professionals. It took a long time to train for example a doctor compared to other professionals (e.g. lawyers); yet, health workers were paid very little salaries compared to other professionals, and also compared to their colleagues in other countries.

d. Lack of facilities at hospitals and health centres. For example, Mbarara hospital started a district hospital. It was upgraded to regional referral hospital and then elevated to the level of a National referral hospital. However, infrastructure, facilitation and human resources (including health workers) were not changed to match the increasing status and demands on the hospital. Available trained health workers were not given adequate and appropriate tools (equipment and drugs) to do their work, were not fully engaged, redundant, demoralised and considered migration.

e. The existing numbers of health workforce was not efficiently utilised, for example, a doctor ran an outpatient clinic in a health centre IV which could be done by a clinical officer and spared the doctor to do theatre work or referred patients with more difficult illnesses clinical officers could not handle.

### Task shifting policy and legal framework

There is no policy or legal framework to underpin task shifting in Uganda. Task shifting is being done informally and haphazardly. World Health Organization provided guidelines and recommendations to guide policy and decision makers, and health workers on the implementation of task shifting. However, respondents argued that it was more expensive to implement task shifting as recommended by World Health Organization than recruiting the already well trained health workers that were currently unemployed.

The argument fronted for no policy on task shifting in Uganda was that once a policy was formulated, its removal would be difficult in the future in case task shifting did not work as was being thought to. Further arguments acknowledged that task shifting was done simply because of the existing health workforce crisis only.

Respondents reflected on their experiences with traditional birth attendants as not encouraging because they delayed pregnant and labouring mothers to consume services provided by well trained health workers. These mothers ended up with deaths of either babies or themselves or both.

Both the clientele and health providers were not protected by law. For example, a nurse or ophthalmic clinical officer doing a cataract surgery punctures the patient’s eyeball and causes blindness was held responsible and could be sued in the courts of law and compensate the patient because the ophthalmic clinical officer was not mandated by any act or professional act to undertake such surgery. The concerned health workers provided health services for which they were not legally mandated to provide and protected.

### Recruitment, deployment and retention of trained health workers

Health workers were recruited into public service by the Health Service Commission and deployed by the Ministry of Health at the national level while at the district level they were recruited by the District Service Commission and deployed by the District Health Officer on behalf of the Chief Administrative Officer.

Respondents acknowledged the presence of many doctors who were unemployed for a long time and eventually got lost from the country to other countries, and some got employed by the private sector (e.g. Non-governmental organizatios) while others took on non-medical professional businesses such as operating shops and farming. There were thousands of nurses and midwives who were unemployed. For example, many nurses holding a Bachelor of Science in Nursing degree and comprehensive nurse training were unemployed partly because no position was created for them in the public service structure, and partly because government has not allocated adequate funds to districts to employ them.

Respondents argued that unemployment, ambiguous career development path or growth or career progression, and unconducive work explained why so many well trained Ugandan health workers migrated to other countries.

Policy and decision makers argued that ‘cadres of health workers in short supply were actually available but government did not have the capacity to employ them. It was argued that health workers were not valued as people who cared for human life and health, and of course health workers were disgruntled in one way or another and demotivated’. In view of the presence of unemployed well trained health workers, respondents argued that there was no need for task shifting until when all the unemployed trained health workers got employed and the shortage still remained.

### Support supervision

Adequate knowledge and skills was required in order to become competent to provide support supervision. However, health workers capable of providing support supervision for shifted tasks were few in numbers and already overwhelmed by heavy workloads and lack of adequate facilitation. So provision of support supervision was not guaranteed once tasks were shifted to other cadres of health workers. Some respondents argued that patients were at risk because delivery of health services was shifted from well qualified to lower cadres of health workers without ensuring continuous support supervision. Other respondents revealed that there was no monitoring or supervisory framework to ensure quality and that was a major problem. There were assumptions that tasks which were delegated were being done well which not always be the case.

### Training of health workers

The training curriculum for the lower ranks in the health profession empowered trainees to provide a limited range of health services.

Continuing medical education (on job training) for health workers in service was offered in Uganda. It empowered health workers with more competencies (knowledge, skills and practices) required to provide improved services and others not previously offered. However, respondents revealed that continuing medical education was not a motivating factor because health workers put in more effort and time to acquire new and improve existing competencies but this did not attract career progression or better benefit package or growing up on the salary scale.

### Nursing aides and village health teams

Nursing Aides (also called nursing assistants) and village health teams do not belong to any of the health professions. They were trained to provide specified basic services and support the health professional. There was no evidence of training curricula for them. However, they were providing more health services than they were mandated. The nursing Aides in some areas manned first level of health system-patient contact facilities (health centre IIs). Nursing aides prescribed and dispensed drugs haphazardly and also administered systemic drugs by injection while village health team members distributed drugs in addition to collecting vital data.

Respondents argued that authorisation of nursing aides and village health team to prescribe and dispense drugs was dangerous to the patients or consumers in terms of abuse (use of inappropriate drugs, poly-pharmacy, and dosages) and development of drug resistance. Other respondents indicated that patients were at risk because task shifting was done without continued support supervision.

Unfortunately, the experiences show that more problems were caused because tasks were shifted without giving proper induction. Respondents acknowledged that training doctors was more expensive for the government than training lower rank health workers such as clinical officers, nurses, mid-wives, public dental health officers, etc. Roles could be delegated to them after induction and ensuring continuous support supervision. Caution must be taken to ensure that qualified health workers do not offload a lot of service to lower cadres at the cost of quality and safety to the health consumers.

### Induction

Available evidence shows that more problems were caused by shifting without giving proper induction. There was little or no adherence to the World Health Organization guidelines and recommendations. Respondents acknowledged that training doctors was more expensive for the government than training lower rank health workers such as clinical officers, nurses, mid-wives, public dental health officers, etc. Roles could be delegated to clinical officers, nurses, mid-wives, public dental health officers, after they have been inducted into their new roles, trained on when to consult and refer patients/clients, and are offered the necessary support supervision regularly after they have been inducted into their new roles, trained on when to consult and refer patients/clients, and are offered the necessary support supervision regularly.

### Opinions about task shifting

Opinions varied with the different key informants. Representatives of the health service consumers revealed that many people in the community were not informed about task shifting. The community was generally not well knowledgeable about the competencies of different health workers and therefore could not differentiate them.

Patients were reportedly uncomfortable and dissatisfied with being handled by low cadre health workers. They wanted to be handled by doctors. They felt bad on learning that the attending health worker was not a doctor. District political leadership questioned why health worker shortages still existed to warrant task shifting when there were many unemployed trained health workers.

Some respondents were of the view that simple procedures (e.g. episiotomy, surgical toilet, incision and drainage of an abscess) could be shifted to or done by mid-wives, nurses, public health dental officer and clinical officer after undergoing a specific training. Allowing a health work to do surgery depended on the complexity of the required procedure.

#### Acceptability of task shifting at policy and decision making level

Task shifting was not documented in national guidelines and policies but it was practiced. Policy and decision makers did not want to officially recognize it. Task shifting was discussed at policy making circles but was not included in the national health policy and guidelines. Policy making circles did not believe in task shifting as the way to go at the moment. It created more problems than it solved. For example, shifting tasks to the clinical officers was based on the premise that clinical officers were more in numbers than doctors, and were the same health workers in primary contact with the outpatients. However, they already had huge volumes of work and task shifting was not the way to go; otherwise it was looking for more problems than could actually be solved.

Take into consideration the dental outpatient visits, it has impacted negatively on oral health indicators. Patients moved from one clinic to another complaining of a similar problem. A respondent said, "I have personally received patients who reported to have visited dental clinic where their teeth were worked on. X-rays were taken and showed substandard work had been done. Information about those clinics and registration status showed that the service providers were not adequately trained and prepared to offer such services".

The quality of dental services especially those offered by providers without formal training e.g. crown and bridge work, orthodontics, root canal therapy and composite restorations was low. Existing evidence (though most of it was undocumented) indicated that the quality of work done by shifting tasks to lower cadres was not of good quality.

Decision and policy makers overwhelmingly believed that there were enough health workers, and that government was in position to train enough health professionals at every rank of healthcare providers, and therefore task shifting was not acceptable in the existing situation.

Task shifting was acceptable and cost-effective solution to health workforce shortages to some of the respondents. A respondent from the NGO sector gave an experience of how task shifting was applied to address the health worker shortage; nursing aides were trained, coached and mentored. These were supplemented with close support supervision and the nursing aides became experts e.g. in setting up intravenous drips, and were reliable. Emphasis was put on ensuring that the recipient cadres received appropriate and focused training on specific delegated health services prior to taking them on.

One respondent said, ‘I believe a doctor has special skills that could not simply be handed over to other health workers. No health consumer liked to be cared for by lower cadre health workers. The patients’ intention was to be attended to by a doctor/physician or surgeon. Many patients accepted to be treated by lower cadre health workers because they did not know the difference between a doctor and clinical officer, once one was dressed in a white gown (clinical coat); they simply took it that they were consulting or being managed by a doctor. So patients ignorantly accepted care from health workers without adequate information about their qualifications and ranks.

#### Challenges faced in task shifting

There were many challenges faced in task shifting, they included;

• The need to identify health workers with the right competences (knowledge, skills and experience). Clinical officers could not be allowed to do a caesarean section. In case of a mistake, the patients could take serious legal actions against them.

• It was resource consuming because it required a lot of time, training and apprenticeship to create confidence, monitoring, and support supervision. The costs involved in task shifting made it more expensive in the long term than recruiting the available and unemployed qualified health workers.

• Highly trained health professionals resisted task shifting. Their arguments were based on the premise that certain procedures could not be shifted to health worker cadres that were not trained to offer them. They would put patients’ lives at risk.

• Those to whom tasks were shifted to usually demanded specific training prior to taking on new roles and a corresponding pay rise or better remuneration which was in most times not given to them.

## Discussion

The shortage of health workers in Uganda has been acknowledged and was worst in hard to reach and stay rural areas. There was a skewed distribution of health workers in favour of urban areas. For example, most doctors (75%) were located or based in urban centres where as about 15-20% of the doctors were located in rural areas where most (about 86%) of the population in Uganda live. Health work shortage in Uganda was characterised by inadequate numbers of health workers, inappropriate skills’ mix, and maldistribution.

Health workers could not be attracted and retained in rural areas partly due to lack of conducive work environment; rewards and sanctions systems. There were no financial incentives and social amenities such as accommodation, well performing schools where children of health staff can obtain education, safe water and electricity supply, and availability of adequate drugs, equipment, supplies enable smooth delivery of health services.

Instability and high staff turnover/attrition were partly explained by low job satisfaction, and poor terms and conditions of service. Low job satisfaction was linked to inappropriate deployment, low salaries, inadequate support supervision, excessive workload, and poor job security [26]. Health workers were easily attracted to other areas where they were paid better packages such as in the private sector or other countries.

Task shifting has been the main stay of health service delivery in Uganda and practiced over decades. There were mixed arguments in favour and disfavour of task shifting. Major arguments of proponents of task shifting were:

• The less trained/specialised health workers were already performing the roles of highly trained/specialised health workers. What they needed was focused training, support supervision, and a task shifting policy and legal framework to protect them in case of uneventful outcomes occurring during the execution of duties.

• Training a doctor takes a long time (at least 6 years) while a nurse, mid-wife and clinical officer, for example, takes a shorter time (at least 2–3 years). The lower cadres of health workers needed focused training and intensive close support supervision by experienced health workers to ensure that service quality standards are maintained. Then, task shifting would partly address the health worker shortage related problems.

• Experience has shown that lower cadres of health workers provided quality health care if well mentored, coached and support supervised. A respondent gave an example of a nursing assistant who after serving for a long time at a hospital became an expert in fixing intravenous lines on patient whose veins were collapsed and doctors could only do a vein cut down.

Major arguments of those in disfavour of task shifting were;

• World Health Organization provided recommendations and guidelines for task shifting but they are largely geared towards preventive care such as Human Immune Deficiency Virus/Acquired Immune Deficiency Syndrome services. World Health Organization further indicated conditions that should be applied to ensure safe, effective, efficient, equitable and sustainable prior to adopting and/or extending task shifting. It was questionable whether these conditions were feasible and sustainable in the current socio-economic situation Uganda finds herself. Opponents to task shifting argued that it was more expensive to implement task shifting according to World Health Organization guidelines and recommendations than hiring new well trained health workers from the existing unemployed pool.

• The lower cadre of health workers receiving delegated duties were not trained and skilled to offer the delegated roles/or services. They were already overloaded with duties to perform. Assigning them additional roles would compromise the quality of health services, and their productivity.

• Task shifting could be applied in the short run but was prone to cause unintended implications for the uninformed health care services consumers. It was not a sustainable solution to the shortage of health workers.

• The government should instead implement the motivation and retention strategy for human resource for health in totality to influence their migration and reduce shortages.

• Consumers of health services were either uninformed or ignorant about the competences of the health providers and/or had no other options. They were also ignorant about their health legal rights otherwise the cost compensating patients would be high.

• There were many unemployed well trained health workers. Government could employ them, and provide conducive working environment (reward and sanctions) in order to attract and retain health workers.

Some respondents expressed frustration. They said ‘task shifting has been discussed in different forums but its implementation was not yet legalised as evidenced by the absence of a policy and legal framework to underpin it. Basically, too much has been talked but implementation of the outputs of the discussion has proved not feasible for fear that reversing a policy in future would be practically impossible.

## Conclusions

Basing on evidence available in published and gray literature, and the findings of this scoping study, it was evident that there was critical shortage of health workers and that task shifting has been practiced in health service delivery in Uganda for decades. There were many unemployed well trained health workers who could be employed to reduce shortage of health workers and hence need for task shifting. It came out loud and clear that government should do the following to reduce the health work shortage and minimise the need for task shifting.

• Government should hire/recruit the unemployed well trained health workers to enhance the numbers health workers in service to alleviate the health workers shortage, and improve the skills mix.

• Recruitment of health workers should be done centrally because some districts (especially hard to reach and stay districts) do not have the capacity to attract and retain health workers.

• Health trainees sponsored by government should sign a contract with government bonding them to serve a ‘mandatory five years period’ before receiving academic transcripts and certificates, and registering with any health professional bodies.

• Government should implement the motivation and retention strategy for human resources for health in totality. Incentives such as accommodation for health workers at health facilities and salaries enhancement to improve health worker attraction and retention, and reduce the attrition rate should be provided.

• Government should create a conducive work environment; better benefit package, equipment, structural changes, adequate water supply and sanitation facilities, and other social amenities should be made available so that health workers are not redundant and risk migration to other sectors and countries.

Task shifting was implemented in intermittent preventive treatment of malaria in pregnancy, home-based management of malaria, family planning services, Human Immune Deficiency Virus/Acquired Immune Deficiency Syndrome patient assessment and antiretroviral treatment, and treatment of tuberculosis with directly observed short therapy. Task shifting was less promoted in surgical services. Task shifting in surgery was limited to surgical toilet, episiotomies, incision and drainage of abscesses, setting up intravenous drips, anaesthesia, and of recent but not extensively done – medical male circumcision. Therefore, it was imperative to;

• Define the scope of task shifting i.e. health services that could and not be provided by specific cadres of health workers so that they do not move away from delegation to abdication.

• Design clear job descriptions and clear guidelines to enable lower rank health workers to perform their roles to the expectation of the health system and health service consumers.

• Establish a functional and effective referral system. The challenge is the immediate place to refer patients to when unable to handle them.

There was no policy to underpin task shifting and provide both guidance and protection of the health workers involved and health service consumers in case of unplanned events happening in the process. It is done informally and haphazardly. The government was unwilling to put a policy in place. It was recommended that;

• Government puts in place a policy and guidelines to provide guidance and protection to health workers involved in task shifting and the health service consumers if task shifting was deemed a good practice.

Health workers taking up delegated services were not trained to deliver these services. They were given short focused training prior to taking up new assignments. Bearing in mind what it takes to produce a well trained health worker, the short focused training was arguably inadequate. It was observed that even support supervision was erratic, not guaranteed, and unlikely to be effective. It was therefore recommended that;

• Training curricula at health training institutions be reviewed with the objective to cover the subjects in greater depths so that trainees are given more competencies (knowledge and skills) in order to take up a wide range of new roles.

• Regulatory bodies in the Ministry of Education and Sports, and Ministry of Health should do more to define and enforce quality training of non-doctor health trainees, and scope for task shifting.

• Government should enforce provision of continuous and effective support supervision to health workers who take up delegated other duties which were not included in their pre-service training curricula.

## Competing interests

There are no competing interests in this research study. We did not received reimbursements, fees, funding, or salary from an organization that may in any way gain or lose financially from the publication of this manuscript, either now or in the future. I do not hold any stocks or shares in the organization that may in any way gain or lose financially from the publication of this manuscript, either now or in the future. I do not hold or currently applying for any patents relating to the content of the manuscript. I have not received reimbursements, fees, funding, or salary from an organization that holds or has applied for patents relating to the content of the manuscript. I do not have a financial competing interest or any non-financial competing interests (political, personal, religious, ideological, academic, intellectual, commercial or any other) to declare in relation to this manuscript or Non-financial competing interests.

## Authors’ contributions

Both authors read and approved the final manuscript. Dr. SOB (author number 1) was the Principal Investigator. He led the proposal development, data collection, data management and analysis, report writing and writing of this article. He is the main architect of this paper. Dr. AK (author number 2) was the Co-Investigator. He participated in all the stages from proposal development through to writing of report and this paper.

## Pre-publication history

The pre-publication history for this paper can be accessed here:

http://www.biomedcentral.com/1472-6963/14/184/prepub
